# Research in mental health during the pandemic

**DOI:** 10.1192/j.eurpsy.2021.222

**Published:** 2021-08-13

**Authors:** F. Gaughran

**Affiliations:** Dept Of Psychosis Studies, Institute of Psychiatry, Kings College London, London, United Kingdom

## Abstract

**Abstract Body:**

The 2020 coronavirus pandemic sparked sudden change in all spheres of life, not least health services. Across Europe clinical research had to adapt. The virus peaked in different places at different times, with London’s first wave in March-May. The National Institute for Health Research paused all face to face research at NHS and social care sites except for nationally prioritised Urgent Public Health (UHP) Covid-19 studies. The first UPH studies focused on acute Covid-19, largely in physical health settings. Research leaders quickly highlighted the need for high quality research data on the effects of the pandemic on the mental health of the general population, as well as the mental health and neuropsychiatric effects of the virus itself, to allow for the development and evaluation of mitigation strategies. The major UK research funders have resourced this. Once the first wave abated, paused research was restarted according to national prioritisation guidance. In Maudsley we worked closely with research teams to develop strategies to make our research programmes as Covid-19 adaptive as possible, maximising remote interaction with research participants, with robust infection prevention procedures if face to face meetings were necessary. Examples of innovative strategies will be shared. In January 2021 with the more transmissible variant of SARS CoV2, face to face research paused again, except where risk was outweighed by patient benefit in continuing. As patients benefit hugely from research and innovation and have better outcomes if treated in ‘research-active’ hospitals, maintaining access to research opportunities without increasing risk of contracting Covid-19 will be key in coming months.

**Disclosure:**

No significant relationships.

